# Supplementary material for: Automated app-based augmented reality cognitive behavioral therapy for spider phobia: Study protocol for a randomized controlled trial
Source: PLoS One. 2022 Jul 13;17(7):e0271175. doi: 10.1371/journal.pone.0271175 (PMC9278761; doi:10.1371/journal.pone.0271175)
Supplement: S1 File — (PDF) [file pone.0271175.s002.pdf]

RESEARCH PROTOCOL

(October 2019)

PROTOCOL TITLE *'ZeroPhobia: Self-guided app-based CBT for aviophobia and arachnophobia'*

**Protocol ID**	***Not applicable***
**Short title**	***ZeroPhobia: Avio- and Arachnophobia***
**EudraCT number**	***Not applicable***
**Version**	***3***
**Date**	***01-10-2019***
**Project leader**	***Dr. T. Donker, Vrije University Amsterdam, Department of Clinical, Neuro- and Developmental psychology, Section of Clinical Psychology (t.donker@vu.nl)***
**Principal investigator(s) (in Dutch: hoofdonderzoeker/ uitvoerder)**	***Dr. T. Donker, Vrije University Amsterdam, Department of Clinical, Neuro- and Developmental psychology, Section of Clinical Psychology (t.donker@vu.nl)***<br><br>***Dr. Annemieke van Straten, Vrije University Amsterdam, Department of Clinical, Neuro- and Developmental psychology, Section of Clinical Psychology (a.van.straten@vu.nl)***<br><br>***Dr. J. van Gelder, University Twente, Faculty of Behavioural, Management, and Social Sciences (j.vangelder@utwente.nl)***<br><br>***Dr. Marieke Toffolo, Vrije University Amsterdam, Department of Clinical, Neuro- and Developmental psychology, Section of Clinical Psychology (m.b.j.toffolo@vu.nl)***<br><br>***Jamie Rhiannon Fehribach, Msc Vrije University Amsterdam, Department of Clinical, Neuro- and Developmental psychology, Section of Clinical Psychology (j.r.fehribach@student.vu.nl)***

	(j.r.fehribach@student.vu.nl)
Sponsor (in Dutch: verrichter/opdrachtgever)	*Vrije University Amsterdam, Department of Clinical, Neuro-, and Development Psychology, Section of Clinical Psychology.*
Subsidising party	*Nederlandse Organisatie voor Wetenschappelijk Onderzoek (NWO): Aspasia Grant*
Independent expert (s)	Assistant Prof. Dr. Lindy Boyette, University of Amsterdam, Department of Clinical Psychology (l.l.n.j.boyette@uva.nl)
Laboratory sites <*if applicable*>	*Not applicable*
Pharmacy <*if applicable*>	*Not applicable*

PROTOCOL SIGNATURE SHEET

Name	Signature	Date
**Sponsor or legal representative:**<br>**Head of Department:**<br>**Prof. dr. Annemieke van Straten, Head of Section of Clinical Psychology, Vrije Universiteit Amsterdam**		
**[Coordinating Investigator/Project leader/Principal Investigator]:**<br>**Dr Tara Donker, Assistant Professor, Section of Clinical Psychology, Vrije Universiteit Amsterdam**		

TABLE OF CONTENTS

1.INTRODUCTION AND RATIONALE....11
2.OBJECTIVES....13
3.STUDY DESIGN....13
4.STUDY POPULATION....14
4.1Population (base)....14
4.2Inclusion criteria....15
4.3Exclusion criteria....15
4.4Sample size calculation....15
5.TREATMENT OF SUBJECTS....16
5.1Investigational product/treatment....16
5.2Use of co-intervention (if applicable)....18
5.3Escape medication (if applicable)....18
6.INVESTIGATIONAL PRODUCT....19
6.1Name and description of investigational product(s)....19
6.2Summary of findings from non-clinical studies....19
6.3Summary of findings from clinical studies....19
6.4Summary of known and potential risks and benefits....20
6.5Description and justification of route of administration and dosage....20
6.6Dosages, dosage modifications and method of administration....20
6.7Preparation and labelling of Investigational Medicinal Product....20
6.8Drug accountability....20
7.NON-INVESTIGATIONAL PRODUCT....20
7.1Name and description of non-investigational product(s)....20
7.2Summary of findings from non-clinical studies....21
7.3Summary of findings from clinical studies....21
7.4Summary of known and potential risks and benefits....21
7.5Description and justification of route of administration and dosage....21
7.6Dosages, dosage modifications and method of administration....21
7.7Preparation and labelling of Non Investigational Medicinal Product....21
7.8Drug accountability....21
8.METHODS....21
8.1Study parameters/endpoints....21
8.1.1Main study parameter/endpoint....22
8.1.2Secondary study parameters/endpoints (if applicable)....22
8.1.3Other study parameters (if applicable)....23
8.2Randomisation, blinding and treatment allocation....24
8.3Study procedures....25
8.4Withdrawal of individual subjects....28
8.4.1Specific criteria for withdrawal (if applicable)....28
8.5Replacement of individual subjects after withdrawal....29
8.6Follow-up of subjects withdrawn from treatment....29

8.7Premature termination of the study 29
9.SAFETY REPORTING 29
9.1 Temporary halt for reasons of subject safety 29
9.2AEs, SAEs and SUSARs 30
9.2.1Adverse events (AEs) 30
9.2.2Serious adverse events (SAEs) 30
9.2.3Suspected unexpected serious adverse reactions (SUSARs) 31
9.3Annual safety report 30
9.4Follow-up of adverse events 30
9.5[Data Safety Monitoring Board (DSMB) / Safety Committee] 31
10.STATISTICAL ANALYSIS 31
10.1Primary study parameter(s) 31
10.2Secondary study parameter(s) 31
10.3Other study parameters 32
10.4Interim analysis (if applicable) 32
11.ETHICAL CONSIDERATIONS 32
11.1Regulation statement 32
11.2Recruitment and consent 33
11.3Objection by minors or incapacitated subjects (if applicable) 34
11.4Benefits and risks assessment, group relatedness 36
11.5Compensation for injury 36
11.6Incentives (if applicable) 36
12.ADMINISTRATIVE ASPECTS, MONITORING AND PUBLICATION 36
12.1Handling and storage of data and documents 36
12.2Monitoring and Quality Assurance 38
12.3Amendments 38
12.4Annual progress report 38
12.5End of study report 38
12.6Public disclosure and publication policy 39
13.STRUCTURED RISK ANALYSIS 39
13.1Potential issues of concern 39
13.2Synthesis 41
14.REFERENCES 42

LIST OF ABBREVIATIONS AND RELEVANT DEFINITIONS

**ABR**	**General Assessment and Registration form (ABR form), the application form that is required for submission to the accredited Ethics Committee; in Dutch: Algemeen Beoordelings- en Registratieformulier (ABR-formulier)**
**AE**	**Adverse Event**
**AR**	**Adverse Reaction**
**BAI**	**Beck Anxiety Inventory**
**CA**	**Competent Authority**
**CBT**	**Cognitive Behavioural Therapy**
**CCMO**	**Central Committee on Research Involving Human Subjects; in Dutch: Centrale Commissie Mensgebonden Onderzoek**
**CV**	**Curriculum Vitae**
**CEQ**	**Credibility/Expectancy Questionnaire**
**DSMB**	**Data Safety Monitoring Board**
**EU**	**European Union**
**FAM**	**Flight Anxiety Modality questionnaire**
**FAS**	**Flight Anxiety Situations questionnaire**
**FSQ**	**Fear of Spider Questionnaire**
**GCP**	**Good Clinical Practice**
**GDPR**	**General Data Protection Regulation; in Dutch: Algemene Verordening Gegevensbescherming (AVG)**
**IB**	**Investigator's Brochure**
**IC**	**Informed Consent**
**IMP**	**Investigational Medicinal Product**
**IMPD**	**Investigational Medicinal Product Dossier**
**IPQ**	**Igroup Presence Questionnaire**
**METC**	**Medical research ethics committee (MREC); in Dutch: medisch-ethische toetsingscommissie (METC)**
**NEQ**	**Negative Effects Questionnaire**
**(S)AE**	**(Serious) Adverse Event**
**SPC**	**Summary of Product Characteristics; in Dutch: officiële productinformatie IB1-tekst**
**Sponsor**	**The sponsor is the party that commissions the organisation or performance of the research, for example a pharmaceutical**

	company, academic hospital, scientific organisation or investigator. A party that provides funding for a study but does not commission it is not regarded as the sponsor, but referred to as a subsidising party.
SPQ	Spider Phobia Questionnaire
SUS	System Usability Scale
SUSAR	Suspected Unexpected Serious Adverse Reaction
UAVG	Dutch Act on Implementation of the General Data Protection Regulation; in Dutch: Uitvoeringswet AVG
VRET	VRET Virtual Reality Exposure Therapy
VR	Virtual Reality
WMO	Medical Research Involving Human Subjects Act; in Dutch: Wet Medisch-wetenschappelijk Onderzoek met Mensen
WSQ	Web Screening Questionnaire

SUMMARY

Rationale: Specific phobias, such as intense fear of flying, heights, or spiders, are the most common forms of mental health disorders worldwide. Specific phobias have a lengthy history of clinical research and very effective treatment for specific phobias using exposure therapy already exist (Wolitzky-Taylor et al., 2008). However, due to high costs, stigma, and long waiting lists, access to evidence-based therapy is currently limited. Meta-analyses on treatment effectiveness for people suffering from specific phobias have shown that Virtual Reality Exposure Therapy (VRET) is as effective as traditional forms of exposure therapy (Marino et al., 2015; Parsons and Rizzo 2008; Powers and Emmelkamp 2008; Opris et al., 2012). VRET, however, involves relatively high costs and limited accessibility, which make it prohibitive for the larger part of the population. This project capitalizes on novel technology and recent scientific advances to develop an affordable treatment modality that is available for anybody, anywhere. Specifically, we will develop and test ZeroPhobia, a self-help VRET for aviophobia and arachnophobia, delivered through a smartphone application (app) in combination with rudimentary cardboard Virtual Reality (VR) glasses. We hypothesize that ZeroPhobia is effective in reducing anxiety symptoms and is user-friendly.

Objective: To determine (1) the clinical effects of ZeroPhobia to reduce aviophobia and arachnophobia symptoms at post-test, (2) the clinical effects of ZeroPhobia at 3- and 12-month follow-ups, (3) the user-friendliness of the intervention, (4) if the usage intensity and/or (5) the feeling of presence influence the primary outcome of ZeroPhobia, (6) if exposure or evaluating thoughts mediate anxiety outcomes, (7) if guided VRET results in reduced participant dropout rates, and (8) whether or not ability to fantasize influences the effectiveness of VRET.

Study design: This project involves two randomized controlled trial (aviophobia and arachnophobia) with two arms: the intervention condition (ZeroPhobia) and a waitlist control condition.

Study population: For aviophobia n = 96 and for arachnophobia n = 72 individuals (18-65 years) who experience aviophobia or arachnophobia symptoms, respectively, will be recruited from the general population to participate in the trial.

Intervention: The intervention ZeroPhobia is 6-week self-help VRET for aviophobia and arachnophobia that is delivered through a smartphone application (app) in combination with rudimentary cardboard VR goggles. ZeroPhobia includes modules of psychoeducation, case examples, exposure through VR, cognitive techniques, monitoring of symptoms, and relapse prevention. Participants in the waitlist condition will be offered the intervention directly after post-test.

Main study parameters/endpoints: The main study parameters are the post-test differences in phobic anxiety symptoms between the experimental and control condition, and follow-up differences in anxiety symptoms between baseline and follow-up in the experimental condition.

Nature and extent of the burden and risks associated with participation, benefit and group relatedness: The burden of participation consists of completing online baseline questionnaires (15 minutes) and performing the intervention (6 modules lasting 5-20 minutes across 6 weeks and daily VR exposure practice from module/week 3 onwards, recommended 10 minutes daily). In addition, participants will be asked to complete an online post-intervention assessment immediately after completion of the intervention (20 minutes) and at follow-up after 3 and 12 months (20 minutes). There is minimal risk involved and the burden to participants is limited.

1. INTRODUCTION AND RATIONALE

Specific phobias are intense and irrational fears for a particular object or situation that poses no objective threat (Ledoux, 2015). Think, for example, of fear of spiders, heights, needles, or flying. People with a specific phobia recognize that their fear is excessive and unreasonable, yet they are unable to overcome it. The object or situation1 causes the person suffering from the phobia to endure intense anxiety and distress, which can significantly interfere with his/her ability to function in their private and working lives. With a lifetime prevalence nearing 10%, specific phobias stand at the top of the cost hierarchy of all mental disorders (Smit et al., 2008). More than 500,000 people in the Netherlands currently suffer from one or more specific phobias and each year there are 75,000 new cases with a specific phobia diagnosis (De Graaf et al., 2010). The estimated annual societal cost of specific phobias is €168 million (Smit et al., 2008). Due to high treatment costs, long waiting lists, and a general reluctance to seek treatment, access to evidence-based therapy is currently limited. Only around 20% of people in the Netherlands with a specific phobia reported having access to treatment (De Graaf et al., 2010). As specific phobia treatment is not covered by health insurance in the Netherlands anymore, the current percentage of people who receive treatment may be even lower. If left untreated, specific phobias can become chronic and increase the risk of developing other mental disorders, such as anxiety and depression. Given the psychological burden phobias carry, along with increased risk of developing comorbid depressive and anxiety disorders and the heavy economic burden for society (Kessler, 2005), there is a need for affordable and scalable self-help interventions.

Specific phobias have a lengthy history of clinical research and very effective treatment already exists (Wolitzky-Taylor et al., 2008). So-called ‘exposure therapy’ refers to a form treatment in which a person is gradually exposed to the object or situation of his/her fear. Over the past decade, a new type of treatment based on these same principles has emerged making use of virtual reality (VR), rather than exposure ‘in vivo’. In virtual reality exposure therapy (VRET), artificially created, computer-generated environments replace in vivo therapy in order to expose clients to their phobia. Meta-analyses on treatment effectiveness for people suffering from anxiety disorders and specific phobias in particular have shown that VRET is as effective as traditional forms of exposure therapy (e.g. Goncalves et al., 2012; Morina et al., 2015; Parsons and Rizzo, 2008; Powers and Emmelkamp, 2008; Opris et al., 2012) in reducing anxiety and other outcomes, such as depression. In a review of 14 studies (Morina et al., 2015), participants receiving VRET for specific phobia improved significantly on behavioral assessments after VRET from pre- to post-test (aggregated uncontrolled effect size $g = 1.23$) as well as when compared with

waitlist control subjects (*g* = 1.41). Furthermore, there were no significant differences between VRET and exposure in vivo at post-test and follow-up (g = -0.09 and 0.53 respectively). In another meta-analysis of Opris et al. (2012), results demonstrated that VRET has better outcomes than waitlist controls, similar efficacy between VRET and C(B)T, and no difference in dropout rate between VRET and exposure in vivo. This research shows that virtual environments can be usefully employed as substitutes for real-world settings (Slater et al., 2006, 2013). Aside from demonstrated effectiveness for anxiety disorders and specific phobias in particular, VRET has a number of additional advantages over traditional in vivo treatment, such as the possibility to conduct therapy within the confines of the therapist's office, rather than having to go outside. Furthermore, VRET offers more flexibility in terms of sequencing and intensity of treatment and graduating of exposure. That is, people can practice more often and with a larger variety of scenarios compared to in vivo exposure. In spite of its advantages over traditional in vivo therapy, VRET still involves relatively high costs and limited accessibility which make it prohibitive for the larger part of the population. Existing VRET often also requires heavy graphic processing capabilities not found in ordinary computers and mobile devices. Additionally, existing VRETs still require the intervention of a therapist.

Research into mobile apps as a method to intervene for psychiatric disorders are promising (e.g. Donker et al., 2013; Eysenbach et al., 2011; Saeb et al., 2015). App-based mental health interventions based on e.g. CBT principles have shown to be effective in reducing mental health symptoms with within-group and between-group intention-to-treat effect sizes ranging from *d* = 0.29 – 2.28 and 0.01 – 0.48 at post-test and follow-up, respectively in a review of Donker et al. (2013). Advantages are better accessibility and participant retention, real-time progress monitoring, portability, and flexibility. As far as we know, one study has investigated the effectiveness of VRET using a mobile application for fear of spiders. Results showed a reduction in anxiety levels (Piercey et al., 2012). Another study investigating VRET on a smartphone has recently found VRET to be effective at reducing spider phobia symptoms and non-inferior to traditional one-session exposure therapy (Miloff et al., 2019).

Recently, we have explored the feasibility and efficacy of ZeroPhobia: Acrophobia, a VRET delivered through a smart phone app using Google cardboards for fear of heights (Donker et al., 2019). The randomized controlled trial (RCT) demonstrated large effect sizes and ZeroPhobia: Acrophobia was rated user-friendly. The aim of the proposed project is to test a (similar) low cost, scalable, and evidence-based solution for aviophobia and arachnophobia symptoms through exposure therapy by integrating VR technology with a smart phone app in two separate RCTs. Both interventions will be tested for its effectiveness in reducing anxiety symptoms and user-friendliness. ZeroPhobia: Arachnophobia will differ

from ZeroPhobia: Aviophobia in that it will be a guided VRET, in order to determine whether drop-out rates are lower than previous, unguided trials of ZeroPhobia. The study will be of a randomized controlled trial (RCT) design and will be conducted amongst adults from the general Dutch populations with aviophobia and arachnophobia symptoms.

We hypothesize that both ZeroPhobia applications will effectively reduce anxiety symptoms as well as be rated as user friendly.

2. OBJECTIVES

Primary Objective: To determine the clinical effects (a reduction in anxiety symptoms – aviophobia and arachnophobia at post-test [between the experimental condition and controls]) of ZeroPhobia, and whether effects are sustainable at 3- and 12-month follow-up (a reduction in anxiety symptoms between baseline and follow-up for those in the experimental condition)

Secondary Objective(s): To determine the user-friendliness of ZeroPhobia, if it is effective in reducing depression and anxiety, to determine whether usage intensity and presence in the VR environment influences the effects of ZeroPhobia, to determine whether exposure or evaluating thoughts mediate anxiety outcomes, to determine whether or not guided VRET is related to reduced participant drop-out rates, and whether ability to fantasize influences the effectiveness of VRET.

3. STUDY DESIGN

A randomized controlled design will be carried out, in which the effectiveness and user-friendliness of an online app-based VR self-help treatment 'ZeroPhobia: Aviophobia' and 'ZeroPhobia: Arachnophobia' will be evaluated. In this study, 96 (aviophobia) and 72 (arachnophobia) participants from the Dutch general population will be randomized in 2 separate RCTs over 2 conditions: the experimental condition (ZeroPhobia: Aviophobia or Arachnophobia) and a waitlist control condition. The duration of the intervention will be 6 weeks. Measures will be taken at baseline, directly after the intervention (6 weeks) and at 3- and 12- month follow-ups. All measures will be completed online. Subjects in the wait-list condition will receive the intervention after completion of the post-test. Randomization (block randomization with 6, 8, 10, and 12 blocks) will be performed by an independent researcher. Figure 1 presents the flow chart of the study.

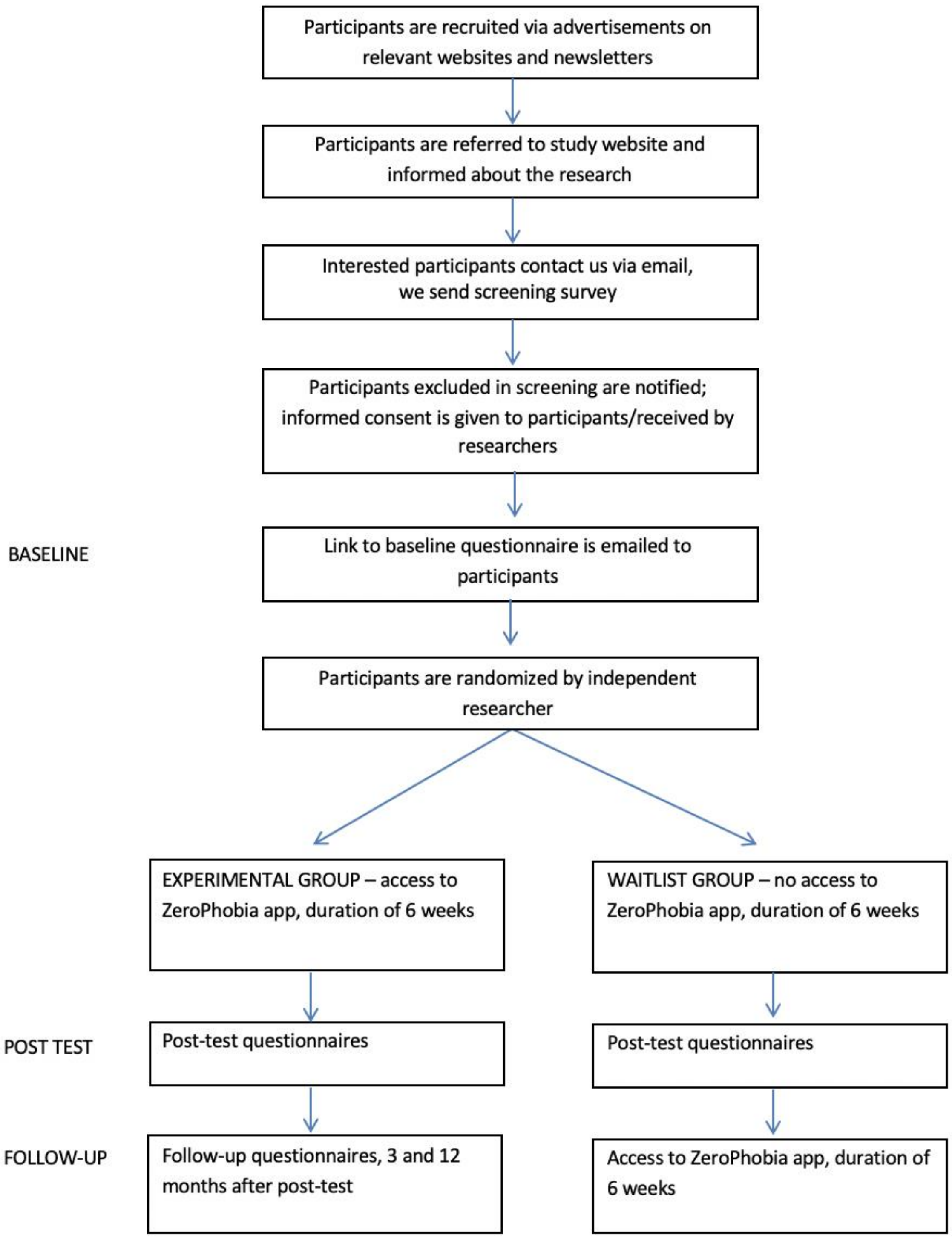

Figure 1: flow chart

4. STUDY POPULATION

4.1 Population (base)

A sample of 96 individuals (18 – 64 years) with symptoms of fear of flying (aviophobia) and 72 individuals with symptoms of fear of spiders (arachnophobia) will be recruited from the Dutch population.

4.2 Inclusion criteria

In order to be eligible to participate in this study, a subject must meet all of the following criteria:

- Aviophobia: scoring above 56 on the Flight Anxiety Situations questionnaire (FAS; van Gerwen et al., 1999; 2018; Nousi et al., 2008)
- Arachnophobia: scoring above 80 on the Fear of Spiders Questionnaire (FSQ; Szymanski et al., 1995)
- between 18 – 64 years old
- have access to a compatible smart phone with internet
- willing to participate in the research study and provide informed consent

Exclusion criteria

A potential subject who meets any of the following criteria will be excluded from participation in this study:

- have insufficient knowledge of the Dutch language
- are under current treatment for specific phobia or psychotropic medication (unless on stable dosage for the previous 3 months and no changes planned during the study period).

4.3 Sample size calculation

Aviophobia: An effect size of Cohen`s d = 0.80 was found from a meta-analysis of VR treatments for fear of flying (Fodor, 2018). Because ZeroPhobia: Aviophobia is a complete self-help treatment with rudimentary VR glasses, we have taken a slightly more conservative effect size of 0.70 as the starting point for the power calculation. To demonstrate a difference between the experimental and control condition with a standardized effect size of d = 0.70 (tested on both sides), an alpha of 0.05 and a statistical power (1-beta) of 0.80, we have 34 respondents in each condition required (68 respondents in total). Taking into account a drop-out rate of 40% (Donker et al., 2019), we need a total of 96 respondents (n = 48 per condition).

Arachnophobia: An effect size of Cohen`s d = 1.11 / 1.12 was found from a meta-analysis of VR treatments for different phobias, including fear of spiders (Opris et al., 2012,

Powers et al., 2008). Because ZeroPhobia: Arachnophobia is a complete self-help treatment with rudimentary VR glasses, we have taken a slightly more conservative effect size of 0.80 as the starting point for the power calculation. To demonstrate a difference between the experimental and control condition with a standardized effect size of d = 0.80 (tested on both sides), an alpha of 0.05 and a statistical power (1-beta) of 0.80, we have 26 respondents in each condition required (52 respondents in total). Taking into account a dropout rate of 40% (Donker et al., 2019), we need a total of 72 respondents (n = 36 per condition).

5. TREATMENT OF SUBJECTS

5.1 Investigational product/treatment

ZeroPhobia is based on the principles of virtual reality exposure therapy (VRET) and Cognitive Behavioral Therapy (CBT). These therapies are currently the most researched and used treatments for anxiety disorders. ZeroPhobia is developed by Vrije Universiteit Amsterdam and In-Session. The content is developed by both, whereas In-Session alone is responsible for app-programming and VR development. ZeroPhobia has been tested by persons with aviophobia symptoms throughout the process of development. In a recent ZeroPhobia user-test, the VR environment was tested in 10 healthy participants. Results showed that the VR environment was rated as realistic and that subjects felt they were present in the environment. Among those who had symptoms of aviophobia, the level of anxiety raised with increased levels of difficulty of the VR environment. Anxiety reduced afterwards. ZeroPhobia: Arachnophobia will be tested among healthy participants soon.

Both ZeroPhobia: Aviophobia as well as ZeroPhobia: Arachnophobia are app-based interventions consisting of six modules that can be followed according to a user's own tempo and timing and without the intervention of a therapist. ZeroPhobia: Aviophobia is entirely unguided, whereas ZeroPhobia: Arachnophobia is guided. The participant will download the app on his or her smartphone. The program can be viewed from their mobile phone. Only when practicing in the VR environment (1 module) participants will put their mobile phone into the provided cardboard VR glasses to be able to experience the VR environment. Participants are allowed to keep the VR glasses after finishing the study.

The therapy consists of six consecutive modules, each taking between 5 to 20 minutes to complete:

1. Psychoeducation: Explanation of a specific phobia and ZeroPhobia
2. Facing your Fears: Setting your goals, explanation about exposure and the fear curve.

3. Exposure Therapy: Practicing in virtual reality and exposure feedback
4. Cognitive Therapy: Detecting automatic thoughts
5. Cognitive Therapy: Developing helping thoughts
6. The Next Steps…..: Facing your fears in daily life

Ad 1) **Psychoeducation**: Explanation of what a specific phobia exactly entails, how it can emerge and what the potential consequences are.

Ad 2) **Facing your fears:** In this module, the participant will define his/her goals for the treatment. Furthermore, the treatment principles underlying exposure are explained.

Ad 3) **Exposure in VR**: in this module, the VR content is explained and how to access it. Participants will practice in a practice-environment with VR before commencing the exposure therapy. After practice, the actual exposure takes place. In the VR environment, participants are exposed to situations involving flying/spiders. Participants can set the level of fear they would like to induce in the environment. Participants are informed that they can remove their VR glasses and exit the exposure at any time, especially if the fear induced is too challenging for them. After completing an exposure and based on his/her fear levels and performance, the user receives feedback. S/he can return to practicing in virtual reality as often as s/he likes and gradually expose him-/herself to different flight or spider situations and fear intensities. Only during the exposure sessions will the participant use the VR glasses.

Ad 4 and 5) **Cognitive therapy**: In these modules, according to the well-established principles of CBT, users identify and evaluate their catastrophic thoughts regarding their fear (e.g., “the airplane will crash” or “the spider will hurt me”).

Ad 6) **The Next Steps**: This module contains information on how the user can continue his/her practice and further reduce his/her fear and prevent relapse. This includes developing an individualized fear hierarchy. A fear hierarchy contains the necessary steps that need to be taken to reach one’s goals in the therapy. Explicit attention is devoted to motivation and encouragement.

The program further consists of:

1) Instructions

Instructions in each of the modules are provided using animated drawings, i.e. 2D animations with a voice-over. The animation underlines the core elements of the therapy.

2) Case examples

The animations contain returning characters who have suffered from aviophobia (Bruno and Chloe) or arachnophobia (Michelle) and had overcome her/his specific phobia. Over the course of the modules this character describes how his/her phobia emerged and developed, what the consequences were for him/her and how s/he overcame it, i.e. through exposure therapy. These characters also provide user feedback and motivate the user to continue with his/her therapy program.

3) Virtual reality

Aviophobia: the prototype immersive VR environment consists of a several flying situations:

- check in and luggage drop-off at the airport
- entering the gateway to the airplane
- take-off
- flying (during day- and night time)
- landing
- turbulence and storm

Arachnophobia: the prototype immersive VR environment consists of a several situations using augmented reality in which different types of spiders (e.g., small, large, thick or thin legs, hairy legs) are projected in the natural environment of the participant, e.g. on a table or on the arm of the participant.

4) Ecological momentary assessment

Participants will be asked about their fear levels before, during and after the exposure VR: *How high is your fear at this moment? Please rate from 0 (no fear) to 10 (very high fear).*

5.2 Use of co-intervention (if applicable)

This study will not interfere with care-as-usual. ZeroPhobia is offered to the participants and can be followed in addition to care as usual if needed.

5.3 Escape medication (if applicable)

Not applicable.

6. INVESTIGATIONAL PRODUCT

6.1 Name and description of investigational product(s)

The ZeroPhobia application is a VR exposure-based intervention for smartphones and consists of six modules, each taking for about 5-20 minutes to complete. The objective of the intervention is to reduce subjects fear of flying or fear of spider symptoms through VR exposure using rudimentary, cardboard VR glasses. At the end of each level, the participant can increase, decrease, or keep the difficulty of the VR environment the same, in such a way that VR exposure is taxed optimally (e.g. not too difficult so that anxiety levels will not raise too high and not too easy so that anxiety levels are too low for exposure to be effective).

6.2 Summary of findings from non-clinical studies

Not applicable. No non-clinical studies have been conducted regarding ZeroPhobia.

6.3 Summary of findings from clinical studies

The working mechanism behind exposure therapy is based on Pavlov`s learning theory of classical condition (Pavlov, 1927; Rescorla & Wagner, 1972). Numerous recent studies show that exposure therapy, virtual reality exposure therapy (VRET) and mobile app-based therapies are effective in reducing symptoms of anxiety.

1) (Meta-analytic) Studies of exposure therapy have repeatedly demonstrated to reduce anxiety levels of specific phobias in different settings (e.g. Kaczkurkin & Foa, 2015; Wolitzky-Taylor et al., 2008). Exposure therapy is one of the most effective treatments (Wolitzky-Taylor et al., 2008).

2) VRET has shown to reduce anxiety and depression levels of participants with specific phobias with similar effectiveness compared to traditional, in-vivo exposure therapy (Goncalves et al., 2012; Krijn et al., 2004; Morina et al., 2015; Opris et al., 2012; Parsons and Rizzo 2008; Powers and Emmelkamp 2008; Raghav et al., 2016; Slater et al., 2006, 2013). Self-help VRET has also shown positive results (e.g. Piercey et al. 2012).

3) Recent research has demonstrated efficacy of mobile app-based therapies for anxiety and depression (Bakker et al., 2016; Donker et al., 2013). Research into mobile apps

as a method to intervene for psychiatric disorders are promising (e.g. Eysenbach et al., 2011; Saeb et al., 2015).

4) Recently we have demonstrated large effect sizes (*d* = 1.14) when ZeroPhobia fear of heights was compared to a waitlist control group (Donker et al., 2019).

The above studies demonstrate the extensive research that has been done on exposure therapy and VRET in particular. The application of exposure therapy as an effective treatment of specific phobia indicates that there is no risk involved in offering VRET as an intervention and that the burden to participants is limited.

6.4 Summary of known and potential risks and benefits

The risks associated with offering ZeroPhobia to individuals who have symptoms of aviophobia or arachnophobia is minimal. In the undue case, participants may experience elevated distress, cyber sickness or fall (see section 13 for a more detailed description regarding the risks). Potential benefits of following ZeroPhobia are a decrease in anxiety symptoms.

6.5 Description and justification of route of administration and dosage

Not applicable.

6.6 Dosages, dosage modifications and method of administration

Not applicable.

6.7 Preparation and labelling of Investigational Medicinal Product

Not applicable.

6.8 Drug accountability

Not applicable.

7. NON-INVESTIGATIONAL PRODUCT

7.1 Name and description of non-investigational product(s)

Not applicable.

7.2 Summary of findings from non-clinical studies

Not applicable.

7.3 Summary of findings from clinical studies

Not applicable.

7.4 Summary of known and potential risks and benefits

Not applicable.

7.5 Description and justification of route of administration and dosage

Not applicable.

7.6 Dosages, dosage modifications and method of administration

Not applicable.

7.7 Preparation and labelling of Non Investigational Medicinal Product

Not applicable.

7.8 Drug accountability

Not applicable.

8. METHODS

8.1 Study parameters/endpoints

8.1.1 Main study parameter/endpoint

Aviophobia: The main parameter will be the 32-item self-report Flight Anxiety Situations questionnaire (FAS; van Gerwen et al., 1999). The questionnaire is frequently used for fear of flying. It has a 5-point Likert scale with an answer format from 1 (no fear) to 5 (overwhelming fear). The questionnaire concerns anxiety related to flying in various aircraft

situations. The FAS has good to excellent reliability and is sensitive in distinguishing people with and without fear of flying (Nousi et al., 2008). The FAS has a 7-point Likert scale (‘not anxious’ to ‘extremely anxious’), with a total possible score between 0 and 120. The questionnaire is widely-used and has good psychometric properties (Cohen, 1977). To be considered for inclusion, individuals have to score above 56.

Arachnophobia: The Fear of Spiders Questionnaire (FSQ, Szymanski & O’Donohue, 1995), an 18-item self-report questionnaire for measuring spider anxiety. Responses can range from 1 to 7, with higher scores indicating more phobic symptoms. Participants can receive a maximum score of 126. This questionnaire is often used for research into spider fear. The FSQ has good to excellent reliability and is sensitive in distinguishing people with and without fear of flying (Szymanski et al., 1995). To be considered for inclusion, individuals must score 80 or above on the FSQ.

These measures will be given at baseline, post-test, and follow-up and each takes less than 5 minutes to complete.

8.1.2 Secondary study parameters/endpoints (if applicable)

- Aviophobia: The self-report Flight Anxiety Modality questionnaire (FAM; van Gerwen et al., 1999) has 18 items measuring anxiety and anticipation anxiety symptoms in flight situations. The FAM has a 5-point Likert answering scale type, from 1 (completely not) to 5 (very intense). The reliability is good (Nousi et al., 2008).
- Questions related to aviophobia: duration of the problem, amount of flights undertaken, safety behaviours, presence of negative flight experiences (at baseline) and questions about whether participants have taken a flight after ZeroPhobia (post-test, follow-up)
- Arachnophobia: The Spider Phobia Questionnaire (Klorman, Hastings, Weerts, Melamed, & Lang, 1974), a 31-item self-report questionnaire for measuring spider anxiety. This questionnaire is often used for research into spider fear. The SPQ has been found to be high in internal consistency (Johnsen & Hugdahl, 1990).
- Beck Anxiety Inventory (BAI; Beck et al., 1988) is a 21-item self-report questionnaire assessing symptoms of anxiety. Patients record how much they have been bothered by each symptom during the past week, including the day the questionnaire is administered. Each item is rated on a 4-point Likert scale ranging from 0 = not at all to 3 = severely: I could barely stand it. The total score ranges from 0 to 63. The following guidelines are recommended for the interpretation of scores: 0 – 9, normal or no

anxiety; 10 – 18, mild to moderate anxiety; 19 – 29, moderate to severe anxiety; and 30 –63, severe anxiety. Internal consistency is high (0.90 – 0.94) and convergent validity is good (Brown et al., 1997).

- The nine-item mood module of the Patient Health Questionnaire (PHQ-9; Kroenke et al, 2007) is used to screen subjects with depressive disorders. The 9 items are each scored 0 – 3, total score range is 0 – 27. In a review of Wittkampf et al. (Wittkampf et al., 2007), a sensitivity of 0.77 (0.71 – 0.84) and a specificity of 0.94 (0.90 – 0.97) was found for the PHQ-9. This questionnaire will be completed at baseline, post-test and follow-up.
- The Web Screening Questionnaire (WSQ; Donker, van Straten, Marks, and Cuijpers, 2009) is a 15-item questionnaire aimed to screen for depressive disorder, alcohol abuse/dependence, GAD, PTSD, social phobia, panic disorder, agoraphobia, specific phobia, and OCD. Only three items of the WSQ will be asked for the purposes of this study – panic disorder, agoraphobia, and OCD. The questionnaire has been found to have both good sensitivity (0.72 – 1.00) and specificity (0.44 – 0.77) (Donker et al., 2009).
- The Interpersonal Reactivity Index (IRI; Davis, 1980) is a self-report questionnaire measuring different types of empathy. 7 items from the sub-section ‘Fantasy’ will be given to participants as baseline. This will be used to measure general ability to fantasized and experience absorption or transportation into fantasy worlds. This questionnaire has been found to be reliable and valid (Davis, 1994).

8.1.3 Other study parameters (if applicable)

- Demographic variables (gender, age, education level, marital status, employment status).
- System Usability Scale (SUS; Bangor et al., 2008): 10 items about user friendliness of the app. The SUS is composed of 10 statements that are scored on a 5-point scale of strength of agreement. Final scores for the SUS can range from 0 to 100, where higher scores indicate better usability. This means that products that are at least passable have SUS scores above 70, with better products scoring in the high 70s to upper 80s. Truly superior products score better than 90. Products with scores less than 70 should be considered candidates for increased scrutiny and continued improvement and should be judged to be marginal at best. Reliability is good (Bangor et al. 2008). This questionnaire will be completed at post-test.
- User-friendliness of ZeroPhobia as a treatment will be in part measured with the 6-item self-report treatment expectation and satisfaction scale called the

Credibility/Expectancy Questionnaire (CEQ; Devilly & Borkovec, 2000). The CEQ measures expectations of the participants before the start of the treatment. This questionnaire will be used alongside the post-test SUS and NEQ to determine if Zero-Phobia did in fact meet expectations.

- User-friendliness will also be measured using single item questions directly asking about the user experience of ZeroPhobia and the realism of the VR environments. This also includes one open question asking for any other feedback on the app a participant would like to give.
- Igroup Presence Questionnaire (IPQ; Schubert, Friedmann and Regenbrecht 2001), a 14-item questionnaire, which assess realism and "presence" in the VR environment. Each of the items has five response categories from fully disagree (1) to fully agree (5). Chronbach`s alpha is good (α = .73). This questionnaire along with some open questions about user experience of the VR environment will be completed at post-test.
- Ecological Momentary Assessment: One question about current anxiety level during exposure in VR
- Usage data from the ZeroPhobia app: frequency and duration of practice during exposure in VR, frequency and duration of time spent in the modules.
- Flight usage questions, including when was the last time you flew on an airplane, how many times have you flown in the past 3/12 months and how long was each of these flights?
- Professional treatment (medication and other psychiatric treatment) will be asked during the screening process, but also at post-test and follow up timepoints in order to control for possible other therapeutic effects.
- Negative Effects Questionnaire (NEQ; Rozental et al., 2016) consists of 32 questions and will be used to asses side effects of psychological treatments. Each question can be answered either 'yes' or 'no.' If yes, participants are then asked to rate how negative the experience of this item was on a 5-point Likert scale (from 'not at all' to 'extremely'). Participants are then asked to determine whether this experience was probably caused by 'the treatment that I am undergoing' or 'other circumstances.' The NEQ has been found to have good validity (Rozental et al., 2019).

8.2 Randomisation, blinding and treatment allocation

The allocation scheme will be created by an independent researcher with a computerized random number generator (Random Allocation Software) with block sizes of 6, 8, 10, and 12 and at an allocation ratio 1:1. Participants will be randomized into two groups: ZeroPhobia or

waitlist condition. Due to the nature of the study, double blinding for treatment allocation is not possible.

8.3 Study procedures

Advertisements with a call to participate in a VR mobile app study for aviophobia and arachnophobia will be posted on several media outlets (Radio, TV), websites (e.g. Fonds Psychische Gezondheid, Angst Fobie en Dwangstichting, Google Adwords, Facebook), mental health institutes (e.g. Stichting Valk for Aviophobia) and in magazines (e.g. de Ingenieur). When a person is interested to participate in the study, s/he can use the URL of the website provided in the advertisement to be directed to the study homepage of ZeroPhobia. On this page, information about the study is provided, including eligibility criteria. If the person thinks s/he fulfills the inclusion criteria and is interested to participate in the study, s/he will be invited to click on a link ('aanmelden'), which will ask them to input their name and email. This information will be sent directly to our secure email inbox, so that we can provide the participants with further study information.

Our website is hosted on one of Vrije Universiteit's Faculty of Movement and Behavior Sciences (FGB) research webservers. This server is located in the server room on the VU campus. Access to this server and room is restricted to system administrators (TO3). Content, such as text, on the website are of the responsibility of the research team. Problems in terms of technical website functioning are the responsibility of TO3.

The website itself is only accessible via the HTTPS protocol and is secured by a SSL-certificate to encrypt all traffic between the web-browser and server. Other than webserver-logs necessary for the webserver, no information about the website visitors is stored on the server.

Information (name and email of the participant) will be received by the researcher, with which we will email more information about the study, specifically the consent form, the participant information letter, and the CMO brochure. In this email, we will ask that if they are still interested in participating after reading these materials, for them to use the provided link to a secure survey.

This survey will allow them to provide a physical mailing address, so hard copies of consent and informational materials can be mailed to them. Screening questions will also include items about age, Dutch fluency, whether or not they are currently receiving psychiatric services for their phobia, and either the FAS or FSQ (duration of 15 minutes). This screener is given prior to official consent in order to save ineligible participants time and prevent unnecessary baseline data from being collected. If participants do not meet inclusion criteria, an automatic email will be sent to them notifying them of their exclusion and the reasoning of such. If participants are

eligible, a trigger email will be sent to the researchers to let us know they meet criteria to participate. This screening information will not be used for any other purposes other than to determine inclusion and exclusion; all screening survey responses will be deleted after participant's eligibility has been determined.

If eligible, according to the basic screener, and still interested, the research assistant will send the patient information letter and informed consent form to their home address with a return envelope. If necessary, the researcher will remind them by email to complete the informed consent (2 reminders in total, second being a phone call). The informed consent form will be sent to each participant. The participant will be instructed to sign the consent form and mail it back to us. Once received, we will sign the consent form as well and then make a copy of the consent form. This copy will be mailed back to the participant to keep for their own records.

Upon receiving informed consent, the researcher will send a second survey link to participants via email to complete a baseline measurement. In this baseline measurement, demographic variables (gender, level of education, marital level), FAM (aviophobia) or SPQ (arachnophobia), PHQ, BAI, WSQ, and IRI will be measured (duration of 20 minutes).

If necessary, the researcher will remind them by email to complete the baseline measurement (2 reminders in total, second being a phone call). After the baseline has been completed, participants will be randomized by an independent researcher. The research assistant will notify the participant of which condition they have been randomized to, either the intervention condition or the waitlist condition.

If in the intervention condition, the research assistant will send the VR glasses to the participant by postal delivery, along with usage instructions. The research assistant will email the participant with instructions on how to download the ZeroPhobia app to his/her smartphone. The app is locked with an individual code which will be given to the participant at the start of the intervention. Participants can then begin the ZeroPhobia intervention which they can follow for 6 weeks (5-20 minutes per week, in addition to VRET from module/week 3 onward, which we recommend 10 minutes a day of practice with). When practicing in VRET, participants are asked to fill in one question about their anxiety level before, during, and after VRET (duration: 5 seconds). Participants can follow the intervention at their own pace. Participants will receive weekly automatic reminder emails to begin or continue the intervention from the research assistant. If needed (e.g., with a question about how to use ZeroPhobia or in the unlikely case of distress), participants will be invited to contact the research assistant.

If in the wait-list condition, the participant will be informed that after a six week pause and the completion of a post-test assessment, they can begin the ZeroPhobia program. The wait-list group will undergo the same program procedure as those in the treatment condition. Participants of both conditions will begin the program on the first working Monday after

completing the baseline and being randomized to a condition, meaning participants of both groups will have different start and end times. Both groups will have the same duration of each section, however (see flowchart).

The aforementioned post-test (occurring after 6 weeks of either intervention or pause, or after the completion of module six in the intervention group (for those participants who choose to work through the ZeroPhobia program faster) consists of the FAS and FAM (aviophobia) or FSQ and SPQ, (arachnophobia), PHQ, BAI, IPQ, SUS, CEQ and NEQ, questions regarding whether they received professional treatment during this time, and questions about flight usage (duration: 25 minutes). Two reminders will be given across two weeks to fill in post-test, the first being an e-mail and the second a phone call.

Those who were randomized to the intervention condition will also receive 3- and 12-month follow-up questionnaires. These surveys will include the FAS and FAM (aviophobia) or FSQ and SPQ (arachnophobia), PHQ, BAI, questions about receiving professional treatment for their phobia, and flight usage questions (duration: 20 minutes). Two reminders will be given across two weeks to fill in follow-up questionnaires, the first being an e-mail and the second a phone call. All assessments are programmed with Survalyzer software. For an overview of measurements, see Table 1. If desired, general information about the outcomes of the study will be sent to the participant after completion of the study.

Table 1: Overview of measures

**Measures**				
	**Aim**	**Baseline**	**Post-test (6w)**	**Follow-up (3, 12 month)**
Demographics	To determine characteristics of sample	x		
FAS or FSQ	To determine symptoms of aviophobia/arachnophobia and treatment effectiveness	x	X	x
FAM or SPQ	To determine symptoms of aviophobia/arachnophobia and treatment effectiveness	x	x	x
BAI	To determine symptoms of anxiety and treatment effectiveness	x	x	x

PHQ	To determine symptoms of depression and treatment effects	x	x	x
SUS	To determine program's user-friendliness		x	
CEQ	To determine program's user-friendliness	x		
NEQ	To determine whether there are any negative effects of ZeroPhobia		x	
IPQ	VR experience		x	
IRI	To determine influence of fantasizing ability on effectiveness of VRET	x		
WSQ	To control for confounding variables	x		
Flight usage	To determine effectiveness of treatment	x	x	x
General flight anxiety questions	To further understand the type of flight anxiety a participant has	x		
User-friendliness and realism questions	To further understand the user experience of the ZeroPhobia app and VR environments		x	
Professional treatment (medication and treatment)	To control for confounding variables	x	x	x

8.4 Withdrawal of individual subjects

Subjects can leave the study at any time for any reason if they wish to do so without any consequences. The investigator can decide to withdraw a subject from the study for urgent medical reasons. In case a participant prematurely terminates his/her participation, s/he will be asked to give permission that the data that were collected until that time point may be included in the ITT-analyses. If the participant refuses to give permission, his/her data will be destroyed.

8.4.1 Specific criteria for withdrawal (if applicable)

Not applicable

8.5 Replacement of individual subjects after withdrawal

Not applicable

8.6 Follow-up of subjects withdrawn from treatment

If a subject is withdrawn from treatment, either due to their own decision or the decision of the research staff, they will still be followed-up with. All participants will be included in the intention-to-treat (ITT)-analyses.

8.7 Premature termination of the study

The study will only be prematurely terminated in case of a serious adverse event (SAE, see 9.1) that is the direct result of the study. In this case the accredited METC and the subsidising party will be informed without undue delay. Participants in either condition will be informed by email, with the content of said email being individual to the specific reason of termination. Participants who are still in the active treatment conditions will receive a notification through email explaining that the study has stopped due to unforeseen circumstances and access to all modules will be blocked. All participants will receive information on where they can seek help for their mental health problems. In addition, the study can be terminated prematurely due to ethical concerns or insufficient participant recruitment.

9. SAFETY REPORTING

9.1 Temporary halt for reasons of subject safety

In accordance to section 10, subsection 4, of the WMO, the sponsor will suspend the study if there is sufficient ground that continuation of the study will jeopardise subject health or safety. The sponsor will notify the accredited METC without undue delay of a temporary halt including the reason for such an action. The study will be suspended pending a further positive decision by the accredited METC. The investigator will take care that all subjects are kept informed.

9.2 AEs, SAEs and SUSARs

9.2.1 Adverse events (AEs)

Adverse events are defined as any undesirable experience occurring to a subject during the study, whether or not considered related to ZeroPhobia. All adverse events reported spontaneously by the subject or observed by the investigator or his staff will be recorded.

9.2.2 Serious adverse events (SAEs)

A serious adverse event is any untoward medical occurrence or effect that

- results in death;
- is life threatening (at the time of the event);
- requires hospitalisation or prolongation of existing inpatients' hospitalisation;
- results in persistent or significant disability or incapacity;
- is a congenital anomaly or birth defect; or
- any other important medical event that did not result in any of the outcomes listed above due to medical or surgical intervention but could have been based upon appropriate judgement by the investigator.

An elective hospital admission will not be considered as a serious adverse event.

The principal investigator (TD) will report all SAEs to the subsidising parties without undue delay after obtaining knowledge of the events. The SAEs will be reported through the web portal *ToetsingOnline* to the accredited METC (VUmc), within 7 days of first knowledge for SAEs that result in death or are life threatening followed by a period of maximum of 8 days to complete the initial preliminary report. All other SAEs will be reported within a period of maximum 15 days after the investigator has first knowledge of the serious adverse events.

9.2.3 Suspected unexpected serious adverse reactions (SUSARs)

Not applicable

9.3 Annual safety report

Not applicable

9.4 Follow-up of adverse events

All AEs will be followed until they have abated, or until a stable situation has been reached. Depending on the event, follow up may require additional tests or medical procedures as indicated, and/or referral to the general physician or a medical specialist. SAEs need to be reported till end of study within the Netherlands, as defined in the protocol.

9.5 [Data Safety Monitoring Board (DSMB) / Safety Committee]

Based on the low risk of participation in the study (see also section 13: Structured Risk Analysis), a data safety monitoring board (DSMB) is not required and will not be set up.

10. STATISTICAL ANALYSIS

10.1 Primary study parameter(s)

The primary outcome measure (FAS for aviophobia, FSQ for arachnophobia) will be treated as a continuous outcome. Continuous variables will be presented as mean, standard deviation, and minimum and maximum number of observations. Descriptive statistics of demographics and clinical outcomes will be compared between the experimental and control condition. The quantitative analysis of the primary endpoints will be performed on an intention-to-treat basis following the per protocol analysis (PPA) according to CONSORT recommendations and SPIRIT guidelines for reporting results (Chan 2013a, 2013b; Eysenbach et al., 2011). Comparisons will be made between and within the groups for pre- and post-measurement and follow-up. Multiple imputation will be used in dealing with missing data, assuming this is "at random" (Brunton-Smith et al., 2014). We will calculate Clinical Meaningful Change on the FAS (Evans, 1998)) and a reliable change criterion (Evans, 1998). We will also calculate the number needed to treat (NNT) based on (Christensen et al., 1986; Cohen, 1972). Standardized effect sizes (Cohen's d) and the confidence intervals are calculated. STATA version 16 will be used for the analyses. The best static analysis procedure to perform the analyses for this RCT will be reviewed before the data is analysed.

Intention-to-treat analysis will be used on continuous scales using repeated measures analysis of variance (ANOVA). Per protocol analysis will be performed by independent sample t-tests. Standardized effect sizes (Cohen's d) and confidence intervals will be calculated. SPSS version 21 will be used for the analyzes. A p-value <0.05 will be considered to indicate statistical significance.

10.2 Secondary study parameter(s)

Continuous variables (FAM or SPQ, PHQ, BAI, IRI) will be presented as mean, standard deviation, and minimum and maximum number of observations. Descriptive statistics of

demographics and clinical outcomes will be compared between the experimental and control condition. The quantitative analysis of the secondary endpoints will be performed on an intention-to-treat basis following the per protocol analysis according to CONSORT recommendations and SPIRIT guidelines for reporting results (Chan 2013a, 2013b; Eysenbach et al., 2011). Comparisons will be made between and within the groups for pre- and post-measurement and follow-up. Fantasizing ability as measured by the IRI will be investigated as a moderating variable for the effectiveness of VRET (on the outcome variable, FAS or FSQ). For the analyses on continuous scales including user data, repeated measures analyses of variance (ANOVA) are used. Multiple imputation will be used in dealing with missing data, assuming this is "at random" (Brunton-Smith et al., 2014). Analyses of flights taken after ZeroPhobia Fear of Flying will be performed using Chi-Square tests to investigate the differences per group. PROCESS (Hayes, 2018) will be used for mediation analysis in SPSS version 25. Standardized effect sizes (Cohen's d) and the confidence intervals are calculated. STATA version 14 will be used for the analyses. The best static analysis procedure to perform the analyses for this RCT will be reviewed before the data is analysed.

10.3 Other study parameters

Continuous variables (IPQ, SUS, CEQ, NEQ) will be presented as mean, standard deviation, and minimum and maximum number of observations. Categorical variables will be presented in terms of frequency numbers and percentages (e.g. demographic variables). Descriptive statistics of demographics and clinical outcomes will be compared between the experimental and control condition. The quantitative analysis will be performed on an intention-to-treat basis following the per protocol analysis according to CONSORT recommendations and SPIRIT guidelines for reporting results (Chan 2013a, 2013b; Eysenbach et al., 2011). For the analyses on continuous scales including user data, repeated measures analyses of variance (ANOVA) are used. Multiple imputation will be used in dealing with missing data, assuming this is "at random" (Brunton-Smith et al., 2014). STATA version 14 will be used for the analyses. The best static analysis procedure to perform the analyses for this RCT will be reviewed before the data is analysed.

10.4 Interim analysis (if applicable)

No interim analyses will be performed.

11. ETHICAL CONSIDERATIONS

11.1 Regulation statement

The study will be conducted according to the principles of the Declaration of Helsinki (World Medical Association, 2013) and in accordance with the Medical Research Involving Human Subjects Act (WMO). Concerning the reporting of results, we will follow the latest version of the Consolidated Standards of Reporting Trials guidelines (CONSORT 2010; Moher et al., 2010). Reports will be provided as outlined under 10.4. The co-principal investigator (AvS) has completed the basic course on regulations and organization for clinical researchers (Dutch: Basiscursus Regelgeving en Organisatie voor Klinisch onderzoekers, BROK®). Other principal investigator, (TD), will be completing this training once courses are once again made available.

11.2 Recruitment and consent

Advertisements with a call to participate in a VR mobile app study for aviophobia/arachnophobia will be shared through several media outlets (Radio, TV), websites (e.g. Fonds Psychische Gezondheid, Angst Fobie en Dwangstichting, Google Adwords, Facebook), mental health institutes (e.g. Stichting Valk for Aviophobia) and in magazines (e.g. de Ingenieur). When a person is interested to participate in the study, s/he can use the URL of the website provided in the advertisement to be directed to the study homepage of ZeroPhobia. On this page, information about the study is provided, including eligibility criteria.

If the person thinks they fulfill the inclusion criteria and is interested to participate in the study, they will be invited to click on a link ('aanmelden'), which opens a new e-mail window with our research team as the addressee. In this way, participants are able to contact us to show interest. Information (name and email of the participant) will be received by the researcher, with which we will email more information about the study, specifically the consent form, the participant information letter, and the CMO brochure. In this email, we will ask that if they are still interested in participating after reading these materials, for them to use the provided link to a secure survey.

This survey will allow them to provide a physical mailing address, so hard copies of consent and informational materials can be mailed to them. Screening questions will also include items about age, Dutch fluency, whether or not they are currently receiving psychiatric services for their phobia, and either the FAS or FSQ (duration of 15 minutes). This screener is given prior to official consent in order to save ineligible participants time and prevent unnecessary baseline data from being collected. If participants do not meet inclusion criteria, an automatic email will be sent to them notifying them of their exclusion and the reasoning of such. If participants are eligible, a trigger email will be sent to the researchers to let us know they meet criteria to

participate. Ineligible participant's screening data will be deleted once ineligibility has been determined.

If eligible and still interested, the research assistant will send the patient information letter and informed consent form to their home address with a return envelope. If necessary, the researcher will remind them by email to complete the informed consent (2 reminders in total). The informed consent form will be sent to each participant. The participant will be instructed to sign the consent form and mail it back to us. Once received, we will sign the consent form as well and then make a copy of the consent form. This copy will be mailed back to the participant to keep for their own records.

Upon receiving informed consent, the researcher will send a second survey link to participants via email to complete a baseline measurement. In this baseline measurement, demographic variables (gender, level of education, marital level), FAS/FAM (aviophobia) or FSQ/SPQ (arachnophobia), PHQ, BAI and WSQ will be measured (duration of 20 minutes).

If necessary, the researcher will remind them by email to complete the baseline measurement (2 reminders in total). After the baseline has been completed, participants will be randomized by an independent researcher. The research assistant will notify the participant of which condition they have been randomized to, either the intervention condition or the waitlist condition.

Objection by minors or incapacitated subjects (if applicable)

Not applicable.

11.3 Benefits and risks assessment, group relatedness

Participants may benefit from their participation in terms of expected reductions in anxiety and depression levels. It is expected that participation will help improve clinical care. Additionally, similar interventions have previously shown to be effective in reducing anxiety and symptoms of specific phobia via VRET through a mobile application (Piercey et al., 2012). Research of another ZeroPhobia app (for fear of heights (acrophobia)) found that self-guided VRET via a smartphone app did effectively reduce specific phobia symptoms (Donker et al., 2019). Furthermore, other research into mobile apps as a method to intervene for psychiatric disorders are promising (e.g. Donker et al., 2013; Eysenbach et al., 2011; Saeb et al., 2015).

Previous studies in similar samples have shown that studies with VRET can safely be carried out, without a significant risk for unwanted effects (e.g. Bouchard et al., 2006; Emmelkamp et al., 2002; Krijn et al., 2004; Piercey et al., 2012). Specifically, previous research on ZeroPhobia: Acrophobia demonstrated that ZeroPhobia can be safely carried out without

serious adverse effects – e.g. no participants reported any injuries during VR use or unbearable anxiety levels during exposure or any other modules of the app (Donker et al., 2019).

One possible risk of participation is the induction of too much fear. In order to treat specific phobia, a certain level of fear is necessary. Without this, treatment would not be effective (Wolitzky-Taylor et al., 2008). In order to assuage the risk of inducing too much fear too quickly, the VR exposure environment is designed to use gradual exposure. This means that subjects start with relatively easy levels of feared situations which induces a small amount of fear. When this situation becomes less fearful, they move on to the next level. In this way, fear levels are manageable. Additionally, participants set their own level of fear induction before beginning an exposure. In this way, they have control over how intense the exposure is, again reducing the risk of inducing too much anxiety.

If a participant does experience extreme distress due to selecting a VR exposure that is too challenging for them, they are instructed to remove their VR glasses. Participants are informed that they are allowed to exit the VR environment or the app at any time, simply by removing their glasses and/or closing the app. In case of an undesirable emotional reaction associated with the modules or VR exposures, the participant is instructed to contact the research assistant, who will inform at least one experienced and licensed clinician (Dr. Donker or Dr. Boyette). This clinician will be available to the participant to provide support if necessary or desired.

Similarly, cybersickness may also occur when using VR technology. Again, participants are informed of this, and told to remove their glasses if this happens. By removing their glasses, levels of high distress or cybersickness are immediately reduced, making it effective in mitigating risk.

Another possible risk associated with this study is a participant feeling imbalanced when using VR technology, causing a risk of falling. To reduce the chance of this occurring, participants are instructed to remove all sharp or otherwise dangerous objects away from their vicinity. They are also told to sit down while using VR. If they do choose to stand, we notify them that they should hold on to something sturdy to help maintain balance. The risk for falling is greater for individuals 65 and older, and for this reason, individuals of this age range are excluded from participating.

A final risk associated with this study is the possibility of questionnaires evoking distress. Questionnaires cover a range of topics, most focused on measuring anxiety symptoms. It is possible that reading and responding to these questionnaires could evoke some distress. However, these instruments are crucial in drawing conclusions about the feasibility and effectiveness of the intervention. In case of an undesirable emotional reaction both during the intervention as well as during the follow up assessments, the participant is instructed to contact

the research assistant, who will inform at least one experienced and licensed clinician (Dr. Donker or Dr. Boyette). This clinician will be available to the participant to provide support if necessary or desired.

If after completion of ZeroPhobia: Fear of Flying participants still have questions about their aviophobia or wish for further treatment, then we will encourage them to contact 'Stichting VALK.' Stichting VALK is an anxiety treatment institute that was created in 1990 in cooperation with University of Leiden, KLM, and Schiphol Airport (www.valk.org; info@valk.org).

Participants will not be withheld regular care during the study; a participant who wishes to seek additional help for their phobia or any other medical issues will not be prevented from doing so.

11.4 Compensation for injury

The VU has a liability insurance which is in accordance with article 7 of the WMO. The VU (also) has an insurance which is in accordance with the legal requirements in the Netherlands (Article 7 WMO). This insurance provides cover for damage to research subjects through injury or death caused by the study.

The insurance applies to the damage that becomes apparent during the study or within 4 years after the end of the study.

11.5 Incentives (if applicable)

No incentives will be provided for taking part in this study. The free provision of treatment is already to the benefit of the participant.

12. ADMINISTRATIVE ASPECTS, MONITORING AND PUBLICATION

12.1 Handling and storage of data and documents

The data will be handled confidentially. All data will be collected at VU University Amsterdam (Section Clinical Psychology) by a staff member. Data (baseline, post-test and follow-up data: name and address, primary and secondary outcome measures) will be captured electronically in a secured online survey platform (Survalyzer B.V., Amsterdam, the Netherlands; www.survalyzer.com). Survalyzer (formerly NetQuestionnaires) is frequently used by university researchers and meets all safety requirements. The data are hosted in an ISO 27001: 2005-certified data center in the Netherlands. With respect to data security, data loss is virtually impossible thanks to an intelligent security system that consists of several steps and a logical wisconcept. The servers are protected against unauthorized access by

biometric access control, video surveillance, and 24-hour physical security. Survalyzer pays specific attention to the general safety risks. The ownership of the data collected by Survalyzer lies solely with the VU.

No personal data will be collected through the ZeroPhobia app. Name, address, telephone numbers, and email addresses will not be collected through the app. Thereby, privacy of participants is protected. Data from the ZeroPhobia app is restricted to anxiety level measures during VR exposure and time spent on and frequency of use of provided modules, including VR and three exercises: setting goals for the intervention, evaluating anxiety provoking thoughts, and creating a personal fear hierarchy.

During the course of the project, this data collected through the app will be stored in a server specifically used for this research. This server will be located at Vrije Universiteit. Disk backups will be timely arranged by the FGB IT department. The backup server and main server are both located at the university. Every night the data will be backed up. This redundant archiving will ensure that data can be recovered in the unlikely event of a critical damage of the primary server. Vrije Universiteit and its servers meet all security standards of the EU. The research-relevant data from the ZeroPhobia app is sent to an API layer (which offers a decoupled interface) using SSL hosted on a server owned and maintained by Vrije Universiteit (zerophobia.labs.vu.nl). All communication between the app and the API is encrypted by means of a certificate. By this, data between the application and the server is secured, because the data is encrypted. The certificate is a guarantee that the app always communicates with the same server, provides two-way authentication, and access can be revoked if deemed necessary. To prevent other app users (non-participants) contaminating the database with data, there are a number of keys defined to validate the addition of new data. Adding new data is only possible if the correct key is sent by one of the participants in the study. This data will then be coded and captured electronically in the secured online database at Vrije Universiteit.

The Vrije Universiteit FGB department is responsible for protection of this data and the virtual server. The data will be uploaded into the IBM SPSS database. All data will be kept in separate databases and merged into a master database only after data collection is completed and each individual database is locked. The data will be coded and linked with the trial identifier consisting of 4 numbers (randomly generated by a random number generator). Data will be coded and the key connecting names to numbers will be kept in a separate, secure location in the principal investigator’s office. Coded data will be electronically stored at Vrije Universiteit, separate from identifying information. Access to data will be password-protected. Only the principal investigators and trial researchers will have access to the final dataset. All collected data will be used only for the purposes of this research. The project group will analyze the data and both positive and negative trial results will be disclosed.

Results will be submitted for publication to peer-reviewed scientific journals. They will be kept for ten years after publication, a policy outlined by de Nederlandse Gedragscode Wetenschapsbeoefening, and will then be destroyed. The publication policy is in agreement with the publication statement of the CCMO (see: www.ccmo.nl).

12.2 Monitoring and Quality Assurance

As participation in this study is of low risk (see also section 13), a monitoring plan will not be needed. The online platform Qualtrics ensures a minimum degree of data quality by allowing certain answers only to be answered in the appropriate format (e.g. in- and exclusion criteria, when asked for their age, participants can only enter numbers etc.).

12.3 Amendments

Amendments are changes made to the research after a favourable opinion by the accredited METC has been given. All amendments will be notified to the METC that gave a favourable opinion.

12.4 Annual progress report

The principal investigator, Tara Donker, will submit a summary of the progress of the trial to the accredited METC once a year. Information will be provided on the date of inclusion of the first subject, numbers of subjects included and numbers of subjects that have completed the trial, serious adverse events/ serious adverse reactions, other problems, and amendments.

12.5 Temporary halt and (prematurely) end of study report

The principal investigator, Tara Donker, will notify the accredited METC of the end of the study within a period of 8 weeks. The end of the study is defined as the last patient's last visit.

The sponsor will notify the METC immediately of a temporary halt of the study, including the reason of such an action.

In case the study is ended prematurely, the sponsor will notify the accredited METC within 15 days, including the reasons for the premature termination.

Within one year after the end of the study, the investigator/sponsor will submit a final

study report with the results of the study, including any publications/abstracts of the study, to the accredited METC.

12.6 Public disclosure and publication policy

The project group will analyze the data, and both positive and negative trial results will be disclosed, unreservedly. Results will be submitted for publication to peer-reviewed scientific journals.

13. STRUCTURED RISK ANALYSIS

13.1 Potential issues of concern

a. Level of knowledge about mechanism of action

The working mechanism behind exposure therapy is based on Pavlov`s learning theory of classical condition (Rescorla & Wagner, 1972). See section 6.3 for an overview of the literature that supports the mechanism behind the ZeroPhobia application.

b. Previous exposure of human beings with the test product(s) and/or products with a similar biological mechanism

ZeroPhobia: Acrophobia, the first study using a mobile app and VR with rudimentary, cardboard VR glasses, showed no deterioration or negative effects, except 24 out of 96 participants experienced 1 or more symptoms of cybersickness. However, because these symptoms overlap completely with anxiety symptoms, this measure may not be a valid measure for cybersickness (Donker et al., 2019).

In a recent user-test of ZeroPhobia: Aviophobia, the VR environment for aviophobia was tested in 10 healthy participants. Results showed that the VR environment was rated as realistic and that subjects felt they were present in the environment. Among those who had symptoms of fear of flying, the level of anxiety raised with increased levels of difficulty of the VR environment. Anxiety reduced afterwards.

A mobile app self-help intervention using VR for spider phobia showed a reduction in anxiety levels (Piercey et al., 2012). In the same line, a number of (meta-analytic) studies have shown that (self-help) VRET reduces anxiety levels in participants with specific phobias (e.g. Krijn et al., 2004; Morina et al., 2015; Parsons and Rizzo 2008; Powers and Emmelkamp 2008; Raghav et al., 2016; Slater et al., 2006, 2013). Furthermore, research into mobile apps as a method to intervene for psychiatric disorders are promising (e.g. Donker et al., 2013; Eysenbach et al., 2011; Saeb et al., 2015).

Extensive meta-analytic research has demonstrated effectiveness of exposure therapy (Kaczkurkin & Foa, 2015; Wolitzky-Taylor et al., 2008). Extensive research has been done regarding the working mechanism, namely classical conditioning (e.g. Bouton, 2016; Brink, 2008; Pavlov, 1927; Rescorla, 1988; Rescorla & Wagner, 1972). These are the key mechanisms of treatment for ZeroPhobia.

c. Can the primary or secondary mechanism be induced in animals and/or in *ex-vivo* human cell material?

No.

d. Selectivity of the mechanism to target tissue in animals and/or human beings

Not applicable.

e. Analysis of potential effect

Participants may benefit from their participation in terms of expected reductions anxiety levels. The risks associated with participation are minimal, whereas benefits are high, as exemplified by previous VRET interventions (Piercey et al., 2012, Donker et al., 2019). Some risks are associated with participating; a description on these risks is given in section 11.3 and 13.1 J of this document.

f. Pharmacokinetic considerations

Not applicable

g. Study population

The study population consists of adult individuals with heightened levels of anxiety for aviophobia or arachnophobia.

h. Interaction with other products

Not applicable

i. Predictability of effect

Not applicable

j. Can effects be managed?

Participants may experience some anxiety distress during the intervention while practicing in the VR environment. However, this level of anxiety is needed for exposure to be effective (Wolitzky-Taylor et al., 2008). Participants will practice with a hierarchy of fear situations using gradual exposure (from low fear situations to high fear situations) in which participants learn to manage their anxiety so their anxiety levels will be tolerable.

Before beginning the exposure, risk effects will be managed by requesting the participant to remove any sharp or otherwise unsafe objects from their vicinity. When beginning treatment, participants are instructed to sit during VR exposures. This is to minimize the risk of falling. However, if the participant does not feel any anxiety during the exposure, they are instructed to stand. This is to eliminate the safety behavior of sitting and to further the effectiveness of the intervention. If standing, participants are told to hold onto a heavy object to further stabilize themselves. Individuals more susceptible to falling and injury due to falls (65 or older) are not eligible to participate in the present study.

Participants may also experience cybersickness during VR exposure. The risk of cybersickness, however, has been minimized in the development of the app itself. Framerate of the videos have been optimized for viewing and there are no fast moving objects in the VR environment. Particiapnts also cannot move quickly through the environment.

Participants are instructed to remover their VR glasses in case they experience cybersickness, high distress, or if they feel out of balance. By removing them, levels of high distress, cyber sickness or out of balance are immediately reduced.

Previous studies in similar samples have shown that studies with VRET can safely be carried out, without a significant risk for unwanted effects (e.g. Bouchard et al., 2006; Emmelkamp et al., 2002; Krijn et al., 2004; Piercey et al., 2012). Furthermore, research into mobile apps as a method to intervene for anxiety disorders is promising (e.g. Eysenbach et al., 2011; Firth., 2017; Saeb et al., 2015). In case of an undesirable emotional reaction during the intervention, the participant can contact the research assistant, who will be available to provide support (including connecting participants to at least one of our team’s clinicians) if necessary or desired.

13.2 Synthesis

Previous studies (see section 6.3) have demonstrated that the working mechanism behind the ZeroPhobia application involves minimal risk. Based on these studies one can assume that offering the ZeroPhobia application to individuals carries low risk. In the undue case of an undesirable emotional reaction, the research assistant and principal investigator/clinician is able to provide adequate support if needed.

A final risk associated with this study is the possibility of questionnaires evoking distress. Questionnaires cover a range of topics, most focused on measuring anxiety symptoms. It is possible that reading and responding to these questionnaires could evoke some distress. However, these instruments are crucial in drawing conclusions about the feasibility and effectiveness of the intervention. In case of an undesirable emotional reaction both during the intervention as well as during the follow up assessments, the participant is instructed to contact the research assistant, who will inform at least one experienced and licensed clinician (Dr. Donker or Dr. Boyette). This clinician will be available to the participant to provide support if necessary or desired.

14. REFERENCES

Abelson JL, Curtis GC. Cardiac and neuroendicrine responses to exposure therapy in height phobics: Desynchrony within the ‘physiological response system.’ *Behaviour Research and Therapy*. 1989;27(5):561–567.

Bakker D, Kazantzis N, Rickwood D, Rickard N. (2016). Mental Health Smartphone Apps: Review and Evidence-Based Recommendations for Future Developments. *JMIR Ment Health.* Mar 1;3(1):e7.

Bangor et al. (2008). An Empirical Evaluation of the System Usability Scale. *International Journal of Human-Computer Interaction*,6, 574-594.

Beck AT, Epstein N, Brown G, et al: An inventory for measuring clinical anxiety: psychometric properties. *J Consult Clin Psychol* 56:893–897, 1988

Brown GK, Beck AT, Newman CF, et al: A comparison of focused and standard cognitive therapy for panic disorder. *Journal of Anxiety Disorders* 11:329–345, 1997

Bouchard S, Cote S, St-Jacques J, et al. Effectiveness of virtual reality exposure in the treatment of arachnophobia using 3D games. *Technol Health Care* 2006; 14:19–27.

Bouton, ME. (2016) Learning and Behavior: A Contemporary Synthesis (2nd ed), Sunderland, MA: Sinauer

Brink, TL. (2008) Psychology: A Student Friendly Approach. "Unit 6: Learning."pp. 97–98

Rescorla, Robert A. Pavlovian Conditioning — It's Not What You Think It Is. (1988) *American Psychologist*, 43, 151–160.

Borkovec TD, Nau SD. Credibility of analogue therapy rationales. Journal of Behavior Therapy and Experimental Psychiatry 1972;3:257–260.

Brunton-Smith IR, Carpenter J, Kenward M, Tarling R. Multiple Imputation for handling missing data in social research. Social Research Update. 2014;65.

Chan A, Tetzlaff J, Altman D, Laupacis A, Gøtzsche P, Krleža-Jerić K, et al. SPIRIT 2013 statement: defining standard protocol items for clinical trials. Ann Intern Med. 2013;158(3):200–7. http://dx.doi.org/10.7326/0003-4819- 158-3-201302050-00583.

Chan A, Tetzlaff J, Gotzsche P, Altman D, Mann H, Berlin J, et al. SPIRIT 2013 explanation and elaboration: guidance for protocols of clinical trials. BMJ. 2013;346:e7586. http://dx.doi.org/10.1136/bmj.e7586.

Christensen, L., Mendoza., JL. A method of assessing changes in as single subject: an alteration of the C Index. Behav Ther. 1986: 17(3): 305-308.

Cohen DC. Personality predictors and the outcome varieties of desensitization. Unpublished doctoral dissertaton. Harvard University, Cambrdige, MA. 972

Coehlo CM, Santos JA, Silvério J, Silva CF. Virtual reality and acrophobia: One-year follow-up and case study. *CyberPsychology and Behavior*. 2006;9(3):336–341.

Devilly; GJ, Borkovec TD. Psychometric properties of the credibility/expectancy questionnaire. Journal of Behavior Therapy and Experimental Psychiatry 2000;31:73–86.

Donker, T., Blankers, M., Hedman, E., Ljotsson, B., Petrie, K., Christensen, H. (2015). Economic evaluations of Internet Interventions: a systematic review. *Psychological Medicine; 4*5 (16), 3357-76.

Donker, T., Cornelisz, I., Van Klaveren, C., Carlbring, P., van Straten, A., Cuijpers, P., Van Gelder, JL. Self-guided app-based virtual reality cognitive behaviour therapy for acrophobia: a randomized clinical trial. (JAMA Psychiatry).

Dutch Smartphone User Report 2015 (2015). http://www.telecompaper.com/research/ dutch-smartphone-user-2015-q3--1113347

Emmelkamp PMG, Bruynzeel M, Drost L, van der Mast CAPG. Virtual reality treatment in acrophobia: A comparison with exposure in vivo. *CyberPsychology and Behavior*. 2001;4(3):335–339.

Eysenbach G. CONSORT-EHEALTH: Improving and Standardizing Evaluation Reports of Web-based and Mobile Health Interventions. J Med Internet Res. 2011;13(4):e126. http://dx.doi.org/10.2196/jmir.1923.

Firth J, Torous J, Nicholas J, Carneya R, Rosenbaum S. (2017): Can smartphone mental health interventions reduce symptoms of anxiety? A meta-analysis of randomized controlled trials. Journal of Affective disorders, 218, 15-22.

Fodor, L. A., Coteț, C. D., Cuijpers, P., Szamoskozi, Ș., David, D., & Cristea, I. A. (2018). The effectiveness of virtual reality based interventions for symptoms of anxiety and depression: A meta-analysis. *Scientific reports*, *8*(1), 10323.

Gerwen Lucas, Spinhoven, P, van Dyck, R., Diekstra, RFW (1999). Construction and psychometric characteristiscs of two self-report questionnaires for the assessment of fear of flying. Psychological Assessment, 11(2): 146-158.

Davis, M.H. (1980). *A multidimensional approach to individual differences in empathy*. Catalog of Selected Documents in Psychology, 10, 85.

Davis, M.H. (1994). *Empathy: A social psychological approach*. Colorado: Westview Press.

De Graaf R, Ten Have M, van Dorsselaer S*. De psychische gezondheid van de Nederlandse bevolking*. Utrecht: Nemesis-2: Opzet en eerste resultaten, Trimbos-Instituut; 2010.

Graaf R de, Have M ten, Gool C van, Dorsselaer S van. Prevalence of mental disorders, and trends from 1996 to 2009. Results from the Netherlands Mental Health Survey and Incidence Study-2 (2012). Social Psychiatry and Psychiatric Epidemiology; 47: 203-213.

Gonçalves, R., Pedrozo A.L., Silva Freire Coutinho E., Figueira I., and Ventura, P. (2012).

Efficacy of Virtual Reality Exposure Therapy in the Treatment of PTSD: A Systematic Review. *PLoS One*.; 7(12): e48469.

Hayes, A. F., Montoya, A. K., & Rockwood, N. J. (2017). The analysis of mechanisms and their contingencies: PROCESS versus structural equation modeling. *Australasian Marketing Journal, 25*, 76-81

Kaczkurkin AN, Foa EB. (2015). Cognitive-behavioral therapy for anxiety disorders: an up-date on the empirical evidence. *Dialogues Clin Neurosci*, 17(3):337-46.

Kessler, R. C., Chiu, W. T., Demler, O., & Walters,E. E. (2005). Prevalence, severity, and comorbidity of 12-month DSM-IV disorders in the National Comorbidity Survey Replication. *Archives of general psychiatry*, 62(6), 617-627.

Krijn M, Emmelkamp PM, Olafsson RP, Biemond R. (2004). Virtual reality exposure therapy of anxiety disorders: a review. *Clin Psychol Rev.* Jul; 24(3):259-81.

Kroenke K, Spitzer RL, Williams JB: The PHQ-9: validity of a brief depression severity measure. *J Gen Intern Med* 2001, 16:606-613.

Ledoux, J. (2015). Anxious. Using the Brain to Understand and Treat Fear and Anxiety. New York: Viking.

Moher, D., Hopewell, S., Schulz, K. F., Montori, V., Gøtzsche, P. C., Devereaux, P. J., . . Altman, D. G. (2010). CONSORT 2010 Explanation and Elaboration: updated guidelines for reporting parallel group randomised trials. *Bmj, 340*. doi:10.1136/bmj.c869

Morina N, Ijntema H, Meyerbröker K, Emmelkamp PM. (2015). Can virtual reality exposure therapy gains be generalized to real-life? A meta-analysis of studies applying behavioral assessments. *Behav Res Ther*.;74:18–24.

Miloff, A., Lindner, P., Dafgård, P., Deak, S., Garke, M., Hamilton, W., Heinsoo, J., Kristoffersson, G., Rafi, J., Sindermark, K., Sjölund, J., Zenger, M., Reuterskiöld, et al. (2019). Automated virtual reality exposure therapy for spider phobia vs. in-vivo one-session treatment: A randomized non-inferiority trial. Behaviour Research and Therapy. 10.1016/j.brat.2019.04.004.

Nousi, A., van Gerwen, L., Spinhoven, P. (2008). The Flight Anxiety Situations Questionnaire and the Flight Anxiety Modality Questionniare: norms for people with fear of flying. Travel Medicine and Infectious Disease, 6, 305-310.

Olatunji BO, Woods CM, de Jong PJ, Teachman BA, Sawchuk CN, David B (2009). Development and initial validation of an abbreviated Spider Phobia Questionnaire using item response theory. Behav Ther. 2009 Jun;40(2):114-30. doi: 10.1016/j.beth.2008.04.002.

Opriş D, Pintea S, García-Palacios A, Botella C, Szamosközi Ş, David D. (2012). Virtual reality exposure therapy in anxiety disorders: a quantitative meta-analysis*. Depress Anxiety;* 29 (2): 85-93.

Parsons, T. D., & Rizzo, A. A. (2008). Affective Outcomes of Virtual Reality Exposure Therapy for Anxiety and Specific Phobias: A Meta-Analysis. *Journal of Behavior Therapy and Experimental Psychiatry,* 39(3), 250-261.
Pavlov, I.P. (1927). *Conditioned Reflexes: An Investigation of the Physiological Activity of the Cerebral Cortex* (translated by G.V. Anrep). London: Oxford University Press.

Piercey CD, Charlton K, Callewaert C (2012). Reducing Anxiety Using Self-Help Virtual Reality Cognitive Behavioral Therapy. *Games Health J,* 1(2):124-8.

Powers, M. B., and Emmelkamp, P. M. (2008). “Virtual Reality Exposure Therapy for Anxiety Disorders: A Meta-analysis”. *Journal of Anxiety Disorders*, 22(3), 561-569.

Raghav K, Van Wijk AJ, Abdullah F, Islam MN, Bernatchez M, De Jongh A. (2016).
Efficacy of virtual reality exposure therapy for treatment of dental phobia: a randomized control trial. *BMC Oral Health*. 16:25.
Reliable and clinically significant changes. Mounted by Chris Evans, 1998.
http://www.psyctc.org/stats/rcsc.htm. Accessed Aug 18, 2018.

Rozental, A., Kottorp, A., Boettcher, J., Andersson, G., & Carlbring, P. (2016). Negative effects of psychological treatments: An exploratory factor analysis of the negative effects questionnaire for monitoring and reporting adverse and unwanted events. *PLoS One*, *11*(6), e0157503.

Rozental, A., Kottorp, A., Forsström, D., Månsson, K., Boettcher, J., Andersson, G., ... & Carlbring, P. (2019). The Negative Effects Questionnaire: psychometric properties of an

instrument for assessing negative effects in psychological treatments. *Behavioural and cognitive psychotherapy*, 1-14.

Rescorla, R. A. & A. R. Wagner (1972). A theory of Pavlovan conditioning: Variations in the effectiveness of reinforcement and nonreinforcement. In *Classical Conditioning II: Current Theory and Research* Black & Prokasy (eds), pp. 64–99. New York: Appleton-Century.

Saeb S, Zhang M, Karr CJ, Schueller SM, Corden ME, Kording KP, Mohr DC. (2015). Mobile Phone Sensor Correlates of Depressive Symptom Severity in Daily-Life Behavior: An Exploratory Study. *J Med Internet Res*. 15;17(7):e175.

Schubert, T., Friedmann, F., & Regenbrecht, H. (2001). The experience of presence: Factor analytic insights. *Presence: Teleoperators and Virtual Environments*, 10, 266-281.

Slater, M., Antley, A., Davison, A., Swapp, D., Guger, C., Barker, C., and SanchezVives, M. V. (2006). "A Virtual Reprise of the Stanley Milgram Obedience Experiments. *PloS one*, 1(1), e39.

Slater, M., Rovira, A., Southern, R., Swapp, D., Zhang, J. J., Campbell, C., and Levine, M. (2013). "Bystander Responses to a Violent Incident in an Immersive Virtual Environment." *PloS one*, 8(1), e52766.

Smit, F., Cuijpers, P., Oostenbrink, J., Batelaan, N., de Graaf, R., Beekman, A. (2006). Costs of nine common mental disorders: implications for curative and preventive psychiatry. *Journal of Mental Health Policy Economics*, 9 (4): 193-200.

Szymanski J, O'Donohue W. Fear of spiders' questionnaire. J Behav Ther Exp Psychiatry. 1995; 26(1): 31–4. pmid:7642758

Wittkampf K, Naeije L, Schene A, Huyser J, Van Weert H: Diagnostic accuracy of the mood module of the Patient Health Questionnaire: a systematic review. *Gen Hosp Psychiat* 2007, 29:388-395.

Wolitzky-Taylor, K.B., Horowitz, J.D., Powers, M.B., Telch, M.J. (2008) .Psychological approaches in the treatment of specific phobias: a meta-analysis*. Clinical Psychology Review,* 28, 1021–1037.

Wolitzky-Taylor, K.B., Horowitz, J.D., Powers, M.B., Telch, M.J. (2008) .Psychological approaches in the treatment of specific phobias: a meta-analysis. Clinical Psychology Review, 28, 1021–1037.
