# Supplementary material for: Automated app-based augmented reality cognitive behavioral therapy for spider phobia: Study protocol for a randomized controlled trial
Source: PLoS One. 2022 Jul 13;17(7):e0271175. doi: 10.1371/journal.pone.0271175 (PMC9278761; doi:10.1371/journal.pone.0271175)
Supplement: S2 File — (PDF) [file pone.0271175.s003.pdf]

Supporting information 3: WHO Trial Registry Data Set

WHO Trial Registry Data Set

Guideline	Information
Registration	Netherlands Trial Registry, NL70238.029.19 (Trial NL9221), 2021-01-26, https://www.trialregister.nl/trial/9221
Funding and Trial Sponsor	Dutch Organization for Scientific Research (Nederlandse Organisatie voor Wetenschappelijk Onderzoek, NWO) via an Aspasia Grant
Contact for Queries	Name: T Donker<br>Email: t.donker@vu.nl<br>Phone: +31 20 5988 265<br>Vrije Universiteit Amsterdam
Public Title	ZeroPhobia: Self-guided app-based Augmented Reality CBT treatment for fear of spider
Scientific Title	ZeroPhobia: Self-guided app-based Augmented Reality CBT treatment for arachnophobia
Country of Recruitment	The Netherlands
Health Condition	Specific Phobia: arachnophobia, fear of spiders
Intervention	**Treatment**: ZeroPhobia is a 6-week, self-help ARET for fear of spiders, that is delivered through a smartphone application. ZeroPhobia includes modules of psychoeducation, case examples, exposure through AR, cognitive techniques, monitoring of symptoms, and relapse prevention.<br><br>**Control**: Our control condition is a waitlist control group. Participants in the waitlist condition will be offered the intervention directly after post-test.
Study Type	1. Interventional<br>2. Allocation: randomized<br>3. Blinding: none<br>4. Assignment: parallel
Date of First Enrollment	Sept 2021
Target Sample Size	150
Recruitment Status	Recruiting

Primary Outcome	Outcome name: Spider fear<br>Method of measurement: The Fear of Spiders Questionnaire (FSQ; Szymanski & O'Donohue, 1995)<br>Timepoints: Screening, post-test, follow-ups
Key Secondary Outcome	Outcome name: Spider fear<br>Method of measurement: The Spider Phobia Questionnaire (SPQ; Klorman et al., 1974)<br>Timepoints: Baseline, post-test, follow-ups
Ethics Review	Status: Approved; October 11th 2019, Medical Ethical Testing Committee (METc), metc@vumc.nl
